# Mild forms of hypophosphatasia mostly result from dominant negative effect of severe alleles or from compound heterozygosity for severe and moderate alleles

**DOI:** 10.1186/1471-2350-10-51

**Published:** 2009-06-06

**Authors:** Delphine Fauvert, Isabelle Brun-Heath, Anne-Sophie Lia-Baldini, Linda Bellazi, Agnès Taillandier, Jean-Louis Serre, Philippe de Mazancourt, Etienne Mornet

**Affiliations:** 1 Unité de pathologie cellulaire et génétique EA2493, Université de Versailles-Saint Quentin en Yvelines, 78035 Versailles, France; 2 Laboratoire SESEP, Centre Hospitalier de Versailles, 78150 Le Chesnay, France; 3 Département de Génie Biologique, Institut Universitaire de Technologies du Limousin, 87000 Limoges, France; 4 Laboratoire de Biomolécules et thérapies antitumorales EA4021, Centre Hospitalier Universitaire de Limoges, Limoges, France

## Abstract

**Background:**

Mild hypophosphatasia (HPP) phenotype may result from *ALPL *gene mutations exhibiting residual alkaline phosphatase activity or from severe heterozygous mutations exhibiting a dominant negative effect. In order to determine the cause of our failure to detect a second mutation by sequencing in patients with mild HPP and carrying on a single heterozygous mutation, we tested the possible dominant effect of 35 mutations carried by these patients.

**Methods:**

We tested the mutations by site-directed mutagenesis. We also genotyped 8 exonic and intronic *ALPL *gene polymorphisms in the patients and in a control group in order to detect the possible existence of a recurrent intronic mild mutation.

**Results:**

We found that most of the tested mutations exhibit a dominant negative effect that may account for the mild HPP phenotype, and that for at least some of the patients, a second mutation in linkage disequilibrium with a particular haplotype could not be ruled out.

**Conclusion:**

Mild HPP results in part from compound heterozygosity for severe and moderate mutations, but also in a large part from heterozygous mutations with a dominant negative effect.

## Background

Loss of function mutations in the *ALPL *gene (MIM 171760) result in hypophosphatasia (HPP) (MIM 146300, 241500, 241510), an inherited disorder characterized by defective bone and teeth mineralization and deficiency of serum and bone alkaline phosphatase (AP) activity [1-3]. The gene codes for tissue-nonspecific alkaline phosphatase (TNSALP), a homodimeric phosphomonoesterase anchored at its carboxyl terminus to the plasma membrane by a phosphatidylinositol-glycan moiety [4,5]. Severe forms of the disease (perinatal and infantile) are transmitted as an autosomal recessive trait while both autosomal recessive and dominant transmission can cause milder forms [1,6-10]. We, along with other groups, have previously showed that *ALPL *gene mutations could exhibit a dominant negative effect leading to a mild HPP phenotype in heterozygotes [11-14]. This dominant negative effect may be due to the inhibition of the activity of the wild-type (WT) monomer by the mutated monomer in heterodimers [11,13,14] or to the sequestration of the WT protein by the mutated one into the Golgi apparatus, preventing it from being transported to the membrane [12]. Carriers of such mutations may not obviously express the disease. In molecular diagnosis, the detection of a single heterozygous mutation in a patient with mild HPP means that a second mutation remains undetected (intronic mutations or mutations in the regulatory sequence) or that the heterozygous mutation has a dominant negative effect. We and others previously showed that the measurement of *in vitro *AP activity in COS cells transfected by the mutated cDNA of *ALPL *correlates with the HPP phenotype [15-21], and that the dominant negative effect of mutations may be tested by co-transfecting mutated and WT cDNAs [13,14,21]. Such a tool may therefore help to distinguish true heterozygosity from compound heterozygosity with an undetected mutation. Indeed, a dominant negative effect of a mutation may indicate true heterozygosity while the absence of such effect may suggest compound heterozygosity, i.e. the existence of another undetected mutation.

In order to evaluate how often we could have missed a second mutation in patients with mild HPP where only one mutation was identified, we tested by site-directed mutagenesis the possible dominant effect of 35 mutations carried by these patients. In addition, we genotyped 8 exonic and intronic *ALPL *gene polymorphisms in the patients and in a control group. This approach should reveal the possible existence of a recurrent undetected intronic mutation detectable by linkage disequilibrium (LD) that could be the cause of the detection failure.

The results show that most of the tested mutations exhibit a dominant negative effect that may account for the mild HPP phenotype of the patients, and for some of the patients, a second mutation in LD with a particular haplotype could not be ruled out.

## Methods

### Patients

Informed consent was obtained from all patients and/or their parents for screening the *ALPL *gene mutations. The study was conducted in compliance with the recommandations of the Comité de Protection des Personnes and with the Helsinki Declaration. In this study mild HPP refers to childhood and adult HPP, odontohypophosphatasia, and perinatal benign HPP, while severe forms are the perinatal and infantile forms. All the patients were unrelated and of apparent European ancestry (Europe, Canada, USA, Australia). We also studied a control group consisting of 74 unrelated patients with severe HPP and carrying two mutations or a homozygous mutation.

### SNPs

All the 8 SNPs studied here are located inside the *ALPL *gene and were genotyped by sequencing during our diagnosis procedure. These SNPs were: c.787T>C (rs3200254), c.793-31C>T, c.862+20G>T (rs2275377), c.862+51G>A (rs2275376), c.862+58C>T (rs2275375), c.863-12C>G, c.863-7T>C and c.876A>G (rs17433807). Haplotyping was determined by parental DNA analysis when available and/or by deduction from the genotype when in the patient one SNP was heterozygotic and the others homozygotic.

### Statistical analysis

Significance of differences between groups of patients was tested by a standard chi-square (χ^2^) test.

### Mutation detection

The primer sequences of the 12 exons were previously reported [22] and allowed analysis of the whole coding sequence, including intron-exon borders and untranslated exons. PCR reactions were performed and analyzed as previously described [22]. The existence of each mutation was confirmed by generating and sequencing a new PCR product obtained independently of the former one, and when possible, by the analysis of parental DNA.

### Transfection studies

A full-length WT cDNA of the *ALPL *gene was obtained by reverse transcription/PCR (RT-PCR) with *Pyrococcus furiosis *DNA polymerase (Stratagene, USA) and standard molecular biology methods. Mutated cDNAs were obtained directly from the normal cloned cDNA by using the Quikchange Site-Directed Mutagenesis kit (Stratagene). The mutated cDNAs were fully sequenced to make sure that the target mutation was inserted and that no other mutation was inserted. Mutated or WT plasmids were transiently transfected in COS-7 cells for 48 hours. Transfections were performed with Lipofectamine and PLUS reagent (Invitrogen) by using the methodology recommended by the manufacturer. The plasmid pcDNA3.1/His/lacZ containing the β-galactosidase gene was used as a positive control of transfection and expression. The cells were treated as previously described [20] and ALP and β-galactosidase activities were determined by monitoring the absorbance at 409 nm and 450 nm respectively. Total AP activity was measured with a COBAS Integra 800 automate (Roche) and was weighted with β galactosidase activity. The pcDNA3.1 vector is designed with a strong promoter so that endogeneous AP activity is neglectible in regard to the inserted gene expression. The quantitative control of transfections and the results were expressed in percent of WT cDNA activity (% WT) taken as reference. For each mutation, experiments were repeated at least four times independently.

The dominant negative effect of mutations was tested by means of co-transfection of COS-7 cells with 50% of WT and 50% of mutant plasmids, according to reference [13]. In these conditions, the absence of dominant negative effect was expected to be 50% WT, while a dominant negative effect was expected to be less than 50%. In this study the threshold between dominant and recessive mutations was set at 40%. This means that mutations between 40 and 50% WT were considered as being without dominant negative effect although they could have a slight dominant negative effect.

### 3D-modeling

A 3D model of the TNAP molecule has been previously constructed by using the homology of TNAP with human placental alkaline phosphatase whose crystal structure has been determined [23,24]. The missense mutations discussed here were localized using the open source PyMOL software .

## Results

Among the 361 samples from unrelated patients sent to our laboratory to explore the possible diagnosis of hypophosphatasia by molecular analysis of the *ALPL *gene, 241 were found to carry at least one single mutation. The other 120 cases were considered to be affected by other pathologies, sometimes identified later, such as campomelic dysplasia or osteogenesis imperfecta. However, we cannot exclude that, despite exhaustive sequencing of the coding sequence and intron/exon borders, some patients were affected with HPP and carried undetected mutations. We detected two mutated alleles in 131 (94.9%) of the 138 severe HPP patients (table 1), a proportion expected for recessively inherited disorders when such a sequencing methodology is used. By contrast only 54 of the 103 patients with mild HPP carried two mutated alleles, and the proportion of detected mutations increased with the severity of the disease (table 1). When tested by site-directed mutagenesis, at least one of the mutations found in these patients produced significant *in vitro *residual AP activity (table 2), and where therefore considered as moderate alleles. The second mutation, when identified, was mostly a severe allele (not shown), indicating that most of these patients were compound heterozygotes for a moderate allele and a severe one. The moderate mutation c.571G>A (p.E191K) was found in 55% of these patients, associated with another mutation, confirming its high frequency in HPP patients from European ancestry [25-27]. For patients with only one heterozygous mutation, clinical symptoms in the parent carrying the mutation were reported in only 30% (14/47) of cases (table 3), mostly loss of teeth in infancy or adulthood, or slight features such as bad teeth, fractures without obvious cause or sagital synostosis at birth. In most of the cases, however, a careful clinical survey of the parents of the patients was not performed.

**Table 1:** Detection rate of *ALPL *mutations in the patients according to their clinical form

**Clinical form**	**One mutation**	**Two mutations**	**Total**
Perinatal	5 (6.4%)	73 (93.6%)	78
Infantile	2 (3.3%)	58 (96.7%)	60
Childhood	8 (23.5%)	26 (76.4%)	34
Adult	12 (40.0%)	18 (60.0%)	30
Odontohypophosphatasia	29 (74.3%)	10 (25.6%)	39

**Total**	**56**	**185**	**241**

The mutations were detected by sequencing the coding sequence, including intron/exon borders, exon 1 and the untranslated part of exon 2.

**Table 2:** The recurrent *ALPL *gene mutations found in the 53 patients affected with mild HPP and carrying two mutated alleles.

**Mutation**	**Activity (% WT)**	**Frequency**
c.571G>A (p.E191K)/other	56–88^a^	0.547
c.526G>A (p.A176T)/other	30.2–45.4^a^	0.094
c.407G>A (p.R136H)/other	33.4	0.075
c.1363G>A (p.G455S)/other	71.0	0.038
c.815G>A (p.R272H)/other	6.8	0.038
c.395C>T (p.A132V)/other	16.9	0.019
other/other		0.189

**Total**		**1.000**

^a ^According to this study and according to Zurutuza et al. [19]

**Table 3:** The mutations found in patients carrying only one heterozygous mutation.

**Patient**	**Mutation**	**Residual activity (%WT)**	**Dominant negative effect (%WT)**	**Parent carrier**	**Clinical symptoms in the parent carrier**	**Haplotypes**	**Trans** **or cis**	**Clin. form**
**1**	p.G456W	0.8	14.5	F	no	A/A	-	Ch
**2**	p.G420A	4.2	19.7	?	no	A/C	-	Od
**3**	p.D378V	1.2	19.9	F	no	A/E	Cis likely	Ch
**4**	p.L414M	0.4	23.5	M	?	A/C	-	Od
**5**	p.P108L	1.9	24.0	M	yes	A/E	trans	Od
**6**	p.N417S	3.0	26.5	M	no	A/A	-	Od
**7**	p.N417S	3.0	26.5	F	yes	A/E	trans	Od
**8**	p.N417S	3.0	26.5	?	no	A/A	-	Ad
**9**	p.N417S	3.0	26.5	F	yes	A/A	-	Od
**10**	p.E452K	1.4	27.5	M	yes	A/B	-	Ad
**11**	p.T100M	1.3	28.2	M	no	A/E	Cis	Ch
**12**	p.R391H	0	29.1	?	?	B/E	Cis	Od
**13**	p.V382I	0^a^	30^a^	F	yes	A/J	-	Pb
**14**	p.R71H	1.0	30.5	?	no	A/B	-	Od
**15**	p.R71H	1.0	30.5	M	?	A/B	-	Od
**16**	p.R71H	1.0	30.5	M	no	A/C	-	Od
**17**	p.R71H	1.0	30.5	M	yes	E/E	Cis/trans	Od
**18**	p.E429K	1.3	31.0	F	no	B/E	Trans	Od
**19**	p.G339R	1.1	33.0	F	no	A/B	-	Od
**20**	p.G339R	1.1	33.0	F	yes	A/B		Od
**21**	p.G339R	1.1	33.0	M	yes	B/E	Trans	Od
**22**	p.R71C	2.5	35.0	F	no	A/A	-	Od
**23**	p.R71C	2.5	35.0	M	no	A/C	-	Od
**24**	p.S445P	2.1	35.1	?	?	A/C	-	Ad
**25**	p.R184W	0.6	36.7	?	?	A/A	-	Ad
**26**	p.R184W	0.6	36.7	M	no	A/A	-	Od
**27**	p.R184W	0.6	36.7	F	yes	A/B	-	Ch
**28**	p.R184W	0.6	36.7	M	no	A/E	Trans	Od
**29**	p.N478I	1.5	38.2	?	no	A/B	-	Ch
**30**	p.G334R	5.0	39.0	?	no	A/C	-	Od
**31**	p.E476A	0.4	39.3	M	?	A/E	Trans	Ad
**32**	p.G334D	1.7	40.0	P	no	A/E	Cis	Od
**33**	p.A116T	0.6	40.0	M	yes	A/E	Trans	Od
**34**	p.I72T	5.1	40.2	M	no	A/B	-	Od
**35**	p.V459L	0	41.1	?	yes	A/A	-	Od
**36**	p.G122S	4.5	43.6	F	?	A/A	-	Od
**37**	p.A111T	2.0	48.7	F	yes	E/E	Cis/trans	Od
**38**	p.R391C	3.75	50.5	M	?	B/E	Trans	Ch
**39**	p.G456R	1.5	50.5	?	no	A/D	-	Ad
**40**	p.G456R	1.5	50.5	?	yes	A/A	-	Od
**41**	p.G456R	1.5	50.5	F	?	A/E	Cis	Ch
**42**	p.A443V	1.38	53.1	?	no	A/A	-	Ad
**43**	p.A176T	30.2	58.0	?	?	A/A	-	Ad
**44**	p.R136H	33.4	70.0	?	?	A/E	-	Ad
**45**	p.G249V	34.5	70.5	F	yes	A/C	-	Ch
**46**	p.I490F	37.1	77.4	?	no	A/E	-	Ad
**47**	p.E191K	56.0	79.5	M	no	A/A	-	Od

The dominant negative effect was measured by co-transfecting wild type (WT) and mutant cDNAs in COS cells and assaying the AP activity. Absence of dominant negative effect is expected to product 50% of WT activity. Parent carrier: F = father; M = mother. Haplotypes correspond to the haplotypes made of the 8 intragenic SNPs studied (see Material and Methods) found in the patient. Trans/Cis indicates the position of the haplotype E regarding to the mutation. Clin. Form = clinical form of the patient.

^a ^As reported by Takinami et al. 2004 [11]

**Figure 1:**
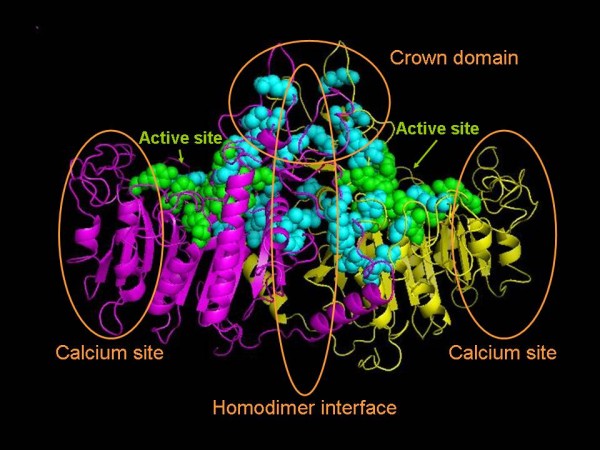
**Location of the dominant mutations on the three-dimensional model of TNAP**. The model is based on the crystal structure of the placental isoform [23]. The two monomers are shown in magenta and yellow. The active site is shown in green and the residues affected by dominant mutations in cyan.

Table 3 summarizes the results of site-directed mutagenesis for 35 mutations found in 47 patients with mild HPP and carrying a single heterozygous mutation. Two mutations were not tested because they corresponded to a frameshift mutation and to a mutation that we failed to introduce in the *ALPL *cDNA by site-directed mutagenesis. Twenty-four of the mutations, representing 36 patients (76.6%) were found to exhibit a dominant negative effect. The dominant negative effect of mutations affecting patients with childhood HPP was slightly stronger (27.9% WT ± 9.2) than patients with odonto and adult HPP (32.5% WT ± 5.9), but the difference was not statistically significant. Interestingly we observed that these mutations were located in particular areas of the 3D model of the protein (figure 1), namely the active site, the crown domain and the homodimer interface. None are located in the calcium site or in the rest of the molecule. The affected domains are clearly involved in allosteric properties of the enzyme and dimerization, and it is therefore understandable to find the mutations with a dominant negative effect in these regions. In order to clarify the cause of the HPP phenotype in the 11 remaining patients carrying mutations with no evidence of a dominant negative effect, we studied the distribution of SNP alleles in the patients and in a control group. The distribution of SNP alleles in the *ALPL *gene showed marked variations from one population to another [28,29]. We therefore considered other HPP patients as the best control group because they were recruited from similar areas and in similar proportions compared to mild HPP patients with one mutation. We also compared our results with the dbSNP  and HapMap  databases when the SNPs tested here were present in those databases. A total of 12 haplotypes were observed, 4 of which were frequent (table 4). This indicates a strong LD between the SNPs of the *ALPL *gene, as previously reported [25,30]. In particular, the markers c.862+20G>T, c.862+51G>A, c.863-12C>G, c.863-7T>C and c.876A>G were found in very strong, almost complete, LD. While other haplotypes showed similar distributions in mild HPP patients and in the control group (Tables 4), the haplotype E, defined by the allelic combination c.787C, c.793-31C, c.862+20T, c.862+51A, c.862+58T, c.863-12G, c.863-7C and c.876G, was found 3 times more frequently in patients with one mutation (p = 1.4 10^-3^). This suggests LD between E and an undetected mutation in a subset of the population carrying this haplotype. Alternatively, a pathogenic effect of the SNP c.862+58T or of a combination of SNPs cannot be ruled out although their high frequencies in various populations [28,29] does not argue for such a pathogenic effect. In order to test this hypothesis we studied by semi-quantitative RT-PCR the expression of the *ALPL *gene in cultured cells of 12 unrelated persons from the general population, 9 carrying E and 9 carrying other haplotypes (not shown). We did not find any difference in regard to splicing and RNA quantity. However, it remains possible that these polymorphisms have an impact on the AP activity itself, which was not tested here (see discussion). In patients where the information was available, we observed that E was in the same proportions in patients with dominant or recessive mutations, and either in trans (on the chromosome that does not carry the mutation) or in cis (on the chromosome that carries the mutation) (Table 3). This was not expected because a disease-causing mutation in LD with E should be more frequent in patients with recessive mutations, and should be found more often in trans.

**Table 4:** Distribution of SNPs haplotypes in the patients carrying only one heterozygous mutation and in patients with two characterized mutated alleles.

Haplotype	Patients with 1 mutation (n = 48)		Other patients (n = 74)		P value
Haplotype A: T-C-G-G-C-C-T-A	54 (0.57)	73 (0.79)	99 (0.67)	137 (0.93)	1.9 10^-1^
				
Haplotype B: T-T-G-G-C-C-T-A	12 (0.13)		23 (0.16)		6.2 10^-1^
				
Haplotype C: T-C-G-G-T-C-T-A	7 (0.07)		5 (0.03)		1.4 10^-1^
				
Haplotype D: T-C-T-A-T-C-T-A	1 (0.01)		0		
				
Haplotype F: T-C-T-A-T-C-T-A	0		2 (0.02)		
				
Haplotype G: T-C-G-G-C-G-T-G	0		2 (0.01)		
				
Haplotype H: C-C-T-A-T-G-T-G	0		1 (0.01)		
				
Haplotype I: C-T-G-G-C-G-C-G	0		1 (0.01)		
				
Haplotype J: C-T-G-G-T-C-T-A	1		1 (0.01)		
				
Haplotype K: C-C-T-A-C-G-C-G	0		2 (0.02)		
				
Haplotype L: T-T-G-G-T-C-T-A	0		2 (0.02)		

Haplotype E: C-C-T-A-T-G-C-G	19 (0.20)	19 (0.21)	10 (0.07)	10 (0.07)	1.4 10^-3^

**Total**	**94**	**94**	**148**	**148**	

Haplotypes are defined by the nucleotide at the SNP locus in the following order: c.787T>C, c.793-31C>T, c.862+20G>T, c.862+51G>A, c.862+58C>T, c.863-12C>G, c.863-7T>C, c.876A>G.

## Discussion

We show here that a large part of mild HPP (i.e. childhood and adult HPP, odontohypophosphatasia, and perinatal benign HPP) is due to heterozygosity for missense mutations with a dominant negative effect, and that the other part is mostly due to compound heterozygosity for mild and severe alleles. The dominant mutations are severe alleles that inhibit the normal monomer when both the normal and the mutated protein form a dimer. The *in vitro *inhibition level varied from 19% WT (p.D378V) to 40% WT (p.I72T) that we chose as the upper limit for a dominant effect. Mutations with activities slightly above this threshold could have a dominant negative effect, but overlapping standard deviations of experimental results made the distinction with recessive mutations impossible. Among the 13 remaining patients, 5 carried mutations with significant residual *in vitro *AP activity: p.A176T (30.2%), p.E191K (56%), p.G249V (34.5%), p.I490F (37.1%) and p.R136H (33.4%). Due to their AP activity and to their frequency in mild HPP patients with two mutations (table 2), p.A176T, p.E191K and p.R136H are clearly mild alleles and it is very likely that the patients carry a second allele not detected by our diagnosis procedure. This is not the case for mutations p.G249V and p.I490F. When co-transfected with the WT allele, the mutation p.G249V does not exhibit a dominant negative effect by inhibition. However we previously showed that this mutation has a dominant negative effect by sequestration of the wild type protein in the Golgi apparatus [12] and thus this mutation should be also classified as dominant Similarly to p.G249V, p.I490F is located on the surface of the molecule, and it is therefore possible that the mutation exhibits a similar effect, but this remains to be demonstrated. The eight other patients carry severe alleles without evidence of an *in vitro *dominant effect. It is therefore possible that these patients harbor a second mutation not detected by sequencing. Due to the mild phenotype of the patient, such a mutation would be a moderate allele, ruling out a large deletion. An intronic mutation or a mutation in the regulatory sequence could account for these patients, in particular a recurrent mutation in LD with haplotype E could affect patients 37, 39 and 41. But the high frequency of E in the group of patients when compared to the control group suggests that E itself, or a mutation in LD, may play a role in the risk for developing mild HPP. We did not demonstrate any effect of the haplotype on *ALPL *RNA expression. However, E may have an effect on the enzyme activity itself. Interestingly the SNP c.787T>C that substitutes tyrosine for histidine at position 263 was previously shown to affect the catalytic property of TNAP and bone mineral density (BMD) in old Japanese women [30,31]. In these studies, the less frequent allele in the Japanese population, allele T, increased the Km and decreased the BMD, and should be therefore considered as the "at risk" allele. By contrast, our study shows that allele C, the less frequent allele in European populations, is associated with haplotype E and should be therefore considered "at risk". The contradiction argues in favor of a causal mutation in LD with C in our HPP patients, rather than in favor of the role of c.787T>C polymorphism itself.

The methodology of cotransfection used here may help the molecular biologist to determine if a patient with mild HPP carries one or two mutated alleles. However, looking at alkaline phosphatase activity in just one cell type, and measuring alkaline phosphatase activity alone should be completed by other studies, especially experiments allowing the detection of abnormalities in intracellular processing and migration, and to distinguish them from changes in catalytic activity on the external surface of cells. Also, it is possible that differences in the RNA expression of the defective allele compared to WT would affect *in vitro *assessment of the mutation. This should be addressed in the future to fully understand phenotype-genotype relationships in hypophosphatasia.

## Conclusion

In conclusion we show here that mild HPP can result from either compound heterozygosity for severe and moderate mutations, but also in a large part from heterozygous mutations with a dominant negative effect. A sequence variation in LD with haplotype E could in addition play the role of an aggravating factor resulting in loss of haplo-sufficiency.

## Competing interests

The authors declare that they have no competing interests.

## Authors' contributions

DF carried out the molecular genetic and transfection studies. IBH and ASLB participated to the design of expression vectors, to cloning and site-directed mutagenesis experiments, and to drafting of the manuscript. LB performed the SNP study. AT participated to sequencing analysis of mutagenized cDNAs and patient's genomic DNA. JLS contributed to statistical analyses. PDM supervised the research group and participated in the design of the study. EM supervised the hypophosphatasia team, conceived of the study, participated in its design and coordination, contributed to the 3D model analysis and to the drafting of the manuscript. All authors read and approved the final manuscript.

## Pre-publication history

The pre-publication history for this paper can be accessed here:


